# Genetic and clinical profile of high myopia patients with rhegmatogenous retinal detachment

**DOI:** 10.3389/fgene.2025.1485874

**Published:** 2025-04-09

**Authors:** Lin Zhou, Farrukhjon Boboev, Hui Chen, Fanwen Jiang, Chun Zhang, Jing Xiao, Hui Jiang, Yongchuan Liao, Zhuping Xu

**Affiliations:** Department of ophthalmology, West China Hospital, Sichuan University, Chengdu, China

**Keywords:** high myopia, rhegmatogenous retinal detachment, RetNet, stickler syndrome, gene ontology, genotype, phenotype

## Abstract

**Introduction:**

Our previous research identified pathogenic variants in RetNet genes in 23.4% of individuals with early-onset high myopia. This study aims to analyze the genetic defects in patients with high myopia complicated by rhegmatogenous retinal detachment.

**Method:**

Whole-exome sequencing was performed on 40 patients with high myopia accompanied by retinal detachment. Variants were filtered from 281 RetNet genes, 178 genes related to syndromic high myopia, 23 non-syndromic high myopia-associated genes, and 29 rhegmatogenous retinal detachment-related genes using a multistep bioinformatics approach. Clinical data were collected for genotype-phenotype correlation analysis.

**Results:**

Pathogenic variants were detected in 47.5% (19/40) in patients with high myopia accompanied by retinal detachment, specifically in RetNet genes (18/40), rhegmatogenous retinal detachment-related genes (11/40), and syndromic high myopia associated genes (10/40). No variants were found in non-syndromic genes. The most prevalent pathogenic genes for high myopia with retinal detachment were Stickler-related genes, including *COL2A1* (10.0%, 4/40) and *COL11A1* (5.0%, 2/40). Patients with Stickler-related gene variants presented the youngest average age of retinal detachment onset (35.17 ± 18.03 years) and shortest axial length (27.63 ± 1.01 mm).

**Conclusion:**

RetNet genes are the predominant causative genes (18/40, 45.0%) in patients with high myopia and retinal detachment. The findings affirm that Stickler syndrome (15%) is a significant etiological factor for high myopia accompanied by retinal detachment. We recommend enhanced comprehensive systemic and ophthalmic examinations for patients with high myopia to enable early detection and prevention of retinal detachment.

## Introduction

High myopia is defined as a refractive error of at least −6.0 diopters accompanied by an axial length exceeding 26.0 mm (Ohno-Matsui et al., 2021). A significant increase in prevalence of myopia has been reported over the past 2 decades, with 30%–50% of adults affected. Moreover, approximately 20% of myopic individuals could progress to high myopia by 2050 (Morris et al., 2021).

The progression of myopia is influenced by a combination of environmental and genetic factors. Early-onset high myopia, defined as myopia of −6.0 diopters or more in early childhood, is often regarded as a monogenic disease (Wang et al., 2022). To date, hundreds of genes and 26 chromosomal loci have been identified as contributors to high myopia through various methods like next-generation sequencing, genome-wide association studies, and twin studies (Govers et al., 2023). Specifically, 23 genes have been identified as associated with nonsyndromic high myopia. Our previous research indicated that 23.4% early onset high myopia cases were associated with RetNet gene (Zhou et al., 2018).

High myopia is strongly associated with an increased risk of ocular complications due to axial elongation. The most common ocular complications of high myopia are open-angle glaucoma, posterior subcapsular cataract, and rhegmatogenous retinal detachment (RRD) (Johnston et al., 2016). RRD, the most common and vision-affecting complication of high myopia, involves the separation of the retinal neuroepithelium from the pigment epithelium (Kwok et al., 2020; Schick et al., 2020). In a large United Kingdom Biobank study, each additional six diopters of myopia increased the risk of retinal detachment by 7.2 times (Han et al., 2020). High myopia was found in 17.0% of eyes with RRD, a prevalence comparable to that in European populations but lower than that in Asian populations, where it reaches 34.0% (Mitry et al., 2011; Li and Beijing, 2003). The annual incidence of RRD is highest among Chinese residents, at 11.6 cases per 100,000 people (Mowatt et al., 2003). Furthermore, 12%–13% of RRD patients exhibit a family history of retinal detachment in their first-degree relatives (Chandra et al., 2015). Evidence of population differences, possibly related to ethnicity, as well as familial clustering, suggests a genetic predisposition to the occurrence of RRD (Wong et al., 1999). To date, 29 genes have been associated with monogenic disorders involving RRD (Govers et al., 2023).

However, the genetic basis of high myopia accompanied by RRD remains largely unexplored. To elucidate the molecular genetics and the phenotype of high myopia with RRD, we recruited 40 patients. Pathogenetic variants of the RetNet genes, genes related to non-syndromic and syndromic high myopia and genes responsible for RRD were filtered using whole exome sequencing. Genotype-phenotype correlation was analyzed based on clinical presentations.

## Methods

### Procedures

Patients with high myopia accompanied by RRD were recruited in our study. High myopia was characterized by a refractive error of less than −6.0 diopters and an axial length greater than 26.0 mm. The probands with high myopia complicated by RRD, along with available family members, were collected. Tractional, exudative, and traumatic retinal detachment, as well as individuals with other ocular or systemic diseases, were excluded. Participants were recruited from the clinic of West China Hospital at Sichuan University, Chengdu, China. The study adhered to the principles of the Declaration of Helsinki and received approval from the Ethics Committee of West China Hospital, Sichuan University. Informed consent was obtained from every participant. This study collected peripheral blood samples along with clinical data, including family history, past medical history, refractive error, best corrected visual acuity (BCVA), and axial length.

### Genotype and phenotype analysis

Genomic DNA was extracted from peripheral blood leukocytes using a QIAamp Blood Midi Kit. Sequencing was performed by DNBSEQ-T7 platform (MGI, Shenzhen, China) with 150 bp paired-end reads. Sanger sequencing was conducted by ABI3730xl sequencer (Applied Biosystems, United States). Post-sequencing, the raw data in FASTQ format underwent quality control to eliminate low-quality reads. Clean reads were then assembled and spliced using the MyGenostics second-generation sequencing analysis platform. Coverage and sequencing quality of the target region were assessed. Pathogenic variants in 281 RetNet genes (https://web.sph.uth.edu/RetNet/), 178 genes related to syndromic high myopia, 23 genes associated with non-syndromic high myopia and 29 genes responsible for RRD were filtered through multi-step bioinformatics analyses as previously reported (Govers et al., 2023; Yi et al., 2022; Jiang et al., 2024) (Supplementary Table S1; Supplementary Figure S1). Sanger sequencing was used to confirm candidate pathogenic variants and conduct co-segregation analysis. The pathogenicity was evaluated according to the American College of Medical Genetics and Genomics (ACMG) genetic variation classification criteria and guidelines (Richards et al., 2015).

### Clinical assessment

Probands with high myopia complicated by RRD underwent ocular examinations by experienced ophthalmologists. A detailed family and ophthalmic history, including duration of low vision and history of refractive or intraocular surgery, was collected. The examinations included assessment of BCVA, refractive error, and axial length. The axial length was measured by an Optical biometer (IOL Master V5.0, Carl Zeiss Meditec AG, Germany).

A genotype-phenotype analysis was conducted on patients harboring different genetic variations. The study focused on three distinct patient groups: (1) individuals carrying known and unknown gene variants, (2) individuals with pathogenic variants in RetNet-associated genes, RRD-related genes, and syndromic high myopia associated genes, (3) individuals with pathogenic variants in Stickler-related genes and other diseases. Comparative analyses were performed on the age of onset, axial length, refractive error, and BCVA among these groups.

### Statistical analysis

The genotypes of patients with high myopia complicated by RRD were compared with those from previous studies involving early-onset and late-onset high myopia using the Chi-square test (χ^2^). Differences in continuous variables, such as BCVA, refractive error, and axial length, were analyzed using independent sample t-tests for comparisons between two groups and one-way ANOVA for comparisons among more than two groups. All statistical analyses were performed using IBM SPSS Statistics 27 (SPSS Inc., Chicago, IL, United States). A p-value of less than 0.05 was considered statistically significant, with all tests being two-sided.

## Results

### Genotype of the patients with high myopia accompanied by RRD

A total of 40 patients suffered high myopia complicated by RRD was recruited in our study. Whole exome sequencing was performed for all of probands. 281 RetNet genes (https://web.sph.uth.edu/RetNet/), 178 genes related to syndromic high myopia, 23 genes associated with non-syndromic high myopia and 29 genes responsible for RRD were analyzed by multistep bioinformatics analyses. A total of 19 probands (47.5%, 19/40) were detected pathogenic variant in these genes (Table 1). Based on multi-step bioinformatics analysis, including nucleotide and protein changes, frequencies from different databases, pathogenicity prediction, and classification according to ACMG guidelines, we identified 19 pathogenic variants. According to ACMG classification, 16 out of 19 variants were classified as likely pathogenic, and 3 out of 19 were classified as pathogenic. Among these, 16 were missense mutations and three were splice site variants. Of the 19 variants, 5 were previously reported, and 14 were novel.

**TABLE 1:** Pathogenic variants detected in patients with high myopia and RRD.

Gene_Symbol	Type	Sample	Type	Position	Exon		Changes		Frequency			
							Nucleotide	Amino_Acid	1000G	ESP6500	gnomAD	ExAC
CAPN5	het	23C702282	RetNet	chr11:76829253	exon8	8	c.1022G > A	p.R341H	—	—	1.19E-05	1.65E-05
COL11A1	het	23C702255	RetNet, Syndromic and RD	chr1:103444283	exon35	35	c.2735C > T	p.P912L	7.99E-04	—	2.27E-04	2.00E-04
COL11A1	het	23C702281	RetNet, Syndromic and RD	chr1:103354445	exon60	60	c.4495C > T	p.P1499S	2.00E-04	—	1.79E-04	9.91E-05
COL2A1	het	23C702250	RetNet, Syndromic and RD	chr12:48378777	exon26	26	c.1626 + 1G > A	—	—	—	**—**	**—**
COL2A1	het	23C702252	RetNet, Syndromic and RD	chr12:48380960	exon20	20	c.1060-1G > C	—	—	—	**—**	**—**
COL2A1	het	23C702270	RetNet, Syndromic and RD	chr12:48370347	exon49	49	c.3439C > G	p.P1147A	—	—	3.98E-06	8.25E-06
COL2A1	het	23C702287	RetNet, Syndromic and RD	chr12:48376314	exon34	34	c.2272G > A	p.A758T	—	—	—	—
EFEMP1	het	23C702251	RetNet	chr2:56097872	exon11	11	c.1303G > A	p.G435R	—	—	3.99E-06	—
FBN1	het	23C702266	Syndromic and RD	chr15:48760167	exon38	38	c.4715C > A	p.T1572N	—	—	—	—
FZD4	het	23C702248	RetNet and RD	chr11:86662209	exon2	2	c.1589G > A	p.G530E	9.98E-04	—	1.67E-04	2.00E-04
IMPDH1	het	23C702283	RetNet	chr7:128034631	exon15	15	c.1573G > A	p.A525T	—	—	3.19E-05	8.26E-06
NR2E3	het	23C702273	RetNet	chr15:72110009	exon9	9	c.1217A > G	p.D406G	—	—	4.03E-06	**—**
PDE6B	het	23C702259	RetNet	chr4:619752-619752	exon1	1	c.337_338insTCCTGGAGGCTT	p.V113delinsVLEAL	—	—	—	**—**
RDH12	het	23C702258	RetNet	chr14:68195905	exon8	8	c.659-3C > G	—	—	—	—	**—**
RP1	het	23C702274	RetNet and Syndromic	chr8:55540769	exon4	4	c.4327C > T	p.R1443W	—	7.70E-05	5.18E-05	6.64E-05
SNRNP200	het	23C702271	RetNet	chr2:96944075	exon39	39	c.5510A > G	p.N1837S	—	—	3.98E-06	-
VCAN	het	23C702263	RetNet, Syndromic and RD	chr5:82837830	exon8	8	c.9008C > T	p.T3003M	—	—	3.21E-05	2.48E-05
VCAN	het	23C702278	RetNet, Syndromic and RD	chr5:82875868	exon14	14	c.9950T > C	p.I3317T	—	—	—	—
LRP5	het	23C702262	RetNet and RD	chr11:68115614	exon2	2	c.391C > T	p.R131C	—	7.70E-05	1.59E-05	8.29E-06

Prediction				Pathogenic_Analysis (ACMG)	Note	Disease
SIFT	PolyPhen_2	MutationTaster	GERP++		(PMID)	
T (0.062)	PD (0.984)	DC (1)	C (4.14)	Likely pathogenic (PM1,PM5, PP2, PP3)	Novel	Vitreoretinopathy, neovascular inflammatory
D (0.043)	B (0.244)	DC (1)	C (5.35)	Likely pathogenic (PS1, PM2, PP3)	23967202	Stickler syndrome
D (0.017)	PD (0.999)	DC (1)	C (5.95)	Likely pathogenic (PS2, PM2, PP3)	Novel	Stickler syndrome
**—**	**—**	DC (1)	C (5.01)	Pathogenic (PVS1, PS1, PM1, PM2, PM4)	10706362; 25525159; 26747767	Stickler syndrome
**—**	**—**	—	—	Pathogenic (PVS1,PS2, PM2,PP3, PP5)	Novel	Stickler syndrome
T (0.445)	B (0.023)	DC (1)	C (5.43)	Likely pathogenic (PM2, PM5, PP3)	Novel	Stickler syndrome
T (0.061)	B (0.006)	DC (1)	C (5.55)	Likely pathogenic (PM2, PM5, PP3, PP4)	Novel	Stickler syndrome
T (0.485)	PD (0.93)	DC (1)	C (4.74)	Likely pathogenic (PS1, PM2, PP3)	26747767	Doyne honeycomb degeneration of retina
T (1)	B (0)	DC (0.93)	C (5.14)	Likely pathogenic (PM1, PM2, PP2 PP3)	Novel	Marfan syndrome
D (0.013)	B (0.078)	DC (1)	C (4.14)	Likely pathogenic (PS1, PM2, PP2 PP3)	25711638; 30097784;30452590;31237656	Exudative vitreoretinopathy 1
D (0)	PD (0.798)	DC (1)	C (4.29)	Likely pathogenic (PM1, PM2, PP2, PP3)	Novel	Leber congenital amaurosis 11
—	PD (0.954)	—	C (5.02)	Likely pathogenic (PS1, PM1, PM2,PP2 PP3)	24891813	Retinitis pigmentosa 37
—	—	—	—	Likely Pathogenic (PM2, PM4, PP1, PP5)	Novel	Night blindness, congenital stationary, autosomal dominant 2
—	—	—	—	Likely Pathogenic (PM2, PM4, PP1, PP5)	Novel	Leber congenital amaurosis 13
D (0)	PD (0.798)	Polymorphism (0.913)	C (4.32)	Pathogenic (PS1, PM2, PM5, PP1, PP3, PP5)	23991373; 31054281; 34426522	Retinitis pigmentosa
T (0.496)	B (0.01)	DC (1)	C (5)	Likely Pathogenic (PM1, PM2, PP1, PP2)	Novel	Retinitis pigmentosa 33
T (0.178)	PD (0.706)	DC (1)	C (5.16)	Likely Pathogenic (PM2, PM5, PP2, PP3)	Novel	Wagner syndrome
D (0.004)	PD (0.813)	DC (1)	C (6.03)	Likely pathogenic (PM1, PM2, PP2, PP3)	Novel	Wagner syndrome
D (0)	PD (0.999)	DC (1)	C (3.71)	Likely pathogenic (PM1, PM2, PP2, PP3)	Novel	Exudative vitreoretinopathy 4

Note: P, Pathogenic; LP, Likely pathogenic; U, Uncertain; T, Tolerated; D, Damaging; PB, Probably_D; B, Benign; DC, Disease_causing; C, Conserved; PVS: very strong pathogenic criterion; PS: strong pathogenic criterion; PM: moderate pathogenic criterion; PP, supporting pathogenic criterion.

**FIGURE 1:**
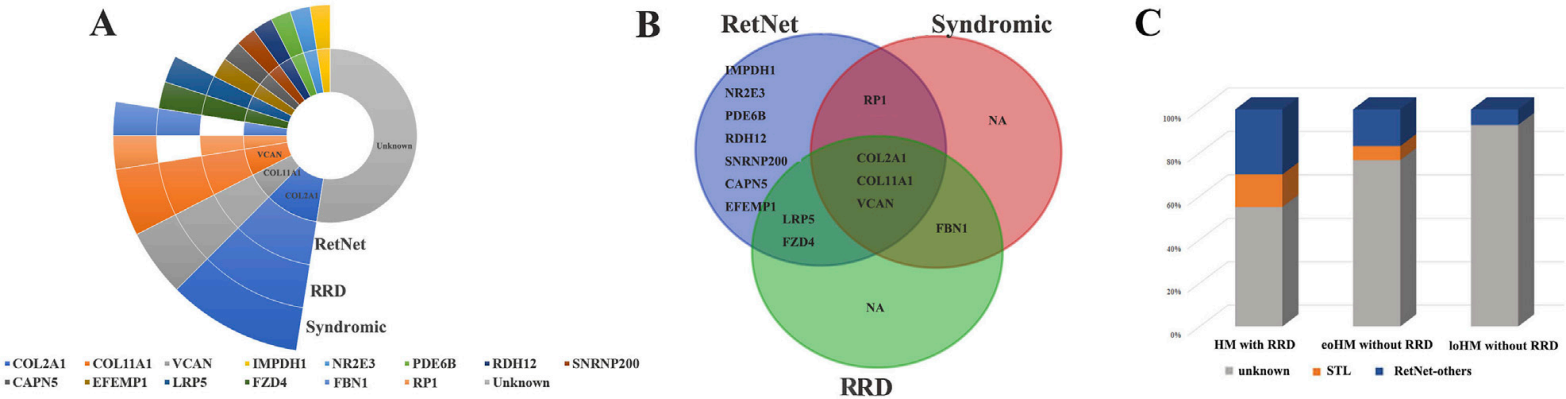
Genotype distribution of high myopia with RRD and comparison with early-onset and late-onset high myopia without RRD in previous studies. **(A)** Distribution of genotypes in high myopia with RRD across different gene categories (including 281 RetNet genes, 178 genes associated with syndromic high myopia, 23 genes linked to non-syndromic high myopia, and 29 genes responsible for rhegmatogenous retinal detachment). The highest proportions of pathogenic genes were found in COL2A1 (4/40, 10.0%), COL11A1 (2/40, 5.0%), and VCAN (2/40, 5.0%). **(B)** Venn diagram showing the distribution of pathogenic genes in high myopia with RD across different groups. **(C)** Comparison of the proportion of high myopia with RD *versus* early-onset high myopia without RD and late-onset high myopia without RD.

The pathogenic variant frequencies in RetNet genes and RRD-related genes and genes associated with syndromic myopia was 45.0% (18/40), 27.5% (11/40) and 25.0% (10/40), respectively. The identified pathogenic variants included 14 genes such as: *COL2A1*, *COL11A1*, *VCAN*, *CAPN5*, *EFEMP1, FBN1*, *FZD4*, *IMPDH1*, *LRP5*, *NR2E3*, *PDE6B*, *RDH12*, *RP1* and *SNRNP200*. Notably, 13 of these 14 pathogenic genes were RetNet genes, with the exception of *FBN1*. *COL11A1*, *COL2A1*, *VCAN*, *FZD4*, *LRP5*, *FBN1* are associated with genes with RRD. *COL11A1*, *COL2A1*, *VCAN*, *FBN1*, *RP1* are linked to syndromic high myopic genes. No pathogenic variants were detected in genes specifically associated with non-syndromic high myopia. The most common pathogenic genes responsible for high myopia with RRD were *COL2A1* (10.0%, 4/40) and *COL11A1* (5.0%, 2/40) and *VCAN* (5.0%, 2/40). *COL2A1* and *COL11A1* were responsible for Stickler syndrome (15%, 6/40) and *VCAN* was associated with Wagner syndrome (5.0%, 2/40) (Table 1; Figure 1).

The pathogenic variants in RetNet genes identified in our study were compared with those in patients with early-onset and late-onset high myopia from our previous studies.

Although the RetNet website now lists 281 genes, an increase from the previous 234, the pathogenic genes identified in our study remain within the original 234 and are not among the newly added genes. Therefore, they are comparable to the previously catalogued genes. More importantly, patients in the previous studies of early-onset and late-onset high myopia did not experience RRD, making the comparison between these two groups feasible. Therefore, a comparison between these two groups is also feasible. The frequency of the variants in RetNet genes in patient with high myopia associated with RRD was significantly higher than early-onset high myopia without RRD (18/40 VS 76/325, 3.18E-3) and late-onset high myopia without RRD (18/40 VS 14/195, 2.11E-10). The frequency of the stickler syndrome related genes in patient with high myopia associated with RRD was significantly higher than late-onset high myopia without RRD (6/40 VS 0/195, 11.70E-5). The frequency in high myopia with RRD was also higher than early-onset high myopia without RRD, although no significant difference was observed (6/40 VS 21/325, 6.00E-2).

### Phenotype of the patients with high myopia accompanied by RRD

**FIGURE 2:**
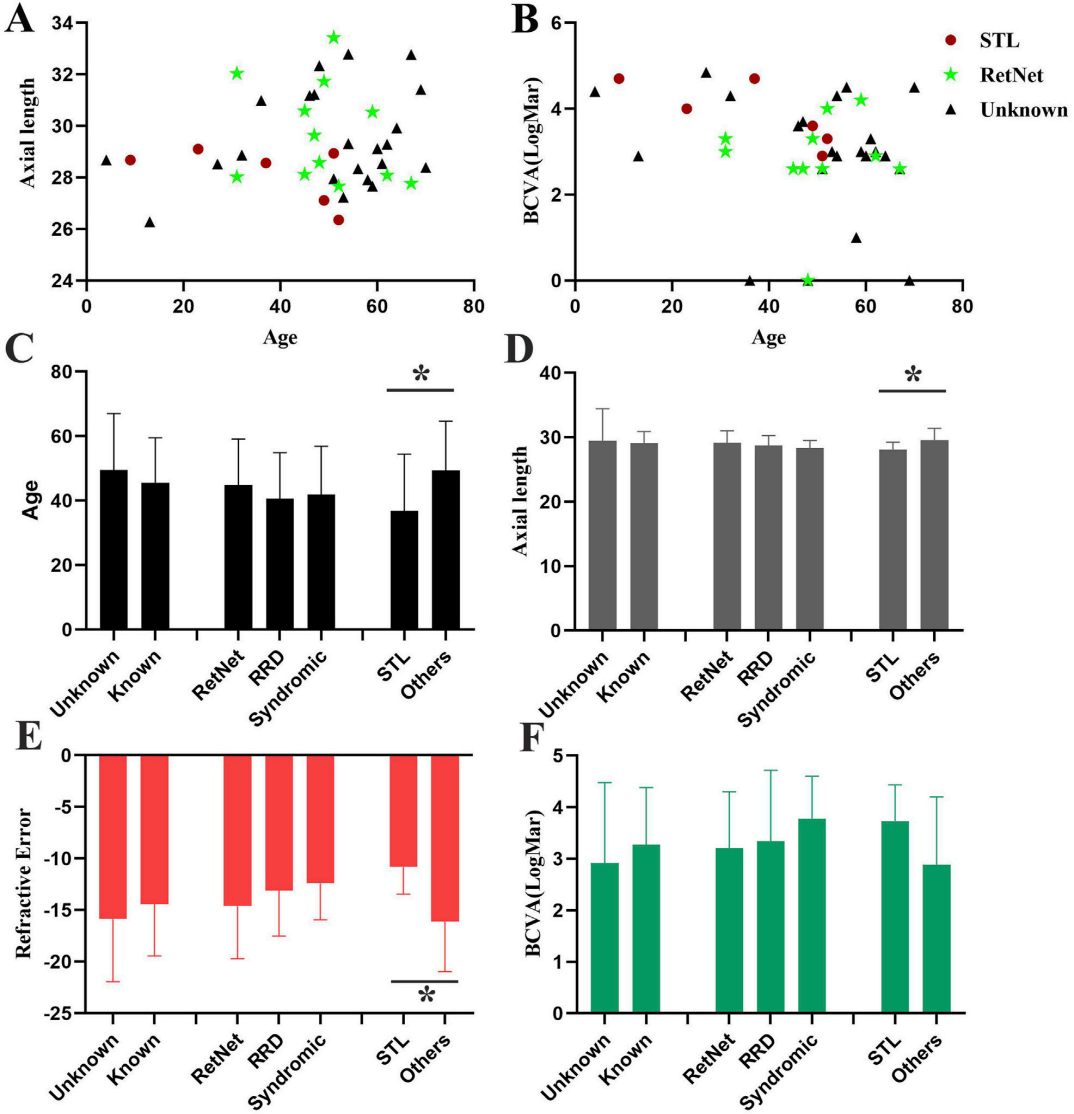
Comparison of phenotypes in patients with high myopia accompanied by RRD. **(A)** Scatter plot showing the distribution of age and axial length for patients with high myopia accompanied by RRD. **(B)** Scatter plot displaying the distribution of age and BCVA for patients with high myopia accompanied by RRD. Red circles indicate patients with Stickler syndrome (STL), green stars represent patients carrying RetNet genes other than STL, and black triangles represent patients with no identified pathogenic genes. **(C–F)**. Comparison of age **(C)**, axial length **(D)**, refractive error **(E)**, and BCVA **(F)** among different patient groups. Three distinct groups are compared: (1) individuals carrying known and unknown gene variants, (2) individuals with pathogenic variants in RetNet-associated genes, RRD-related genes, and syndromic high myopia associated genes, (3) individuals with pathogenic variants in Stickler-related genes and other diseases.

The average age at the onset of RRD was 47.23 ± 16.28 years old, the mean refractive error was −15.30 ± 4.95 diopters, and the average axial length was 29.26 ± 1.87 mm. The best corrected visual acuity of after the surgery of RRD is 3.01 ± 1.28. The onset age of retinal detachment of the patients with pathogenic known variants (45.47 ± 14.02 years old) is lower than patients with pathogenic unknown variants (49.59 ± 17.46 years old), though no significantly difference is detected (Figure 2).

No significant differences were observed in refractive error, axial length, or BCVA after retinal detachment surgery between patients with known and unknown pathogenic variants, or among patients with variants in RetNet genes, RRD-related genes, and syndromic high myopia genes. However, significant difference was found between patients with pathogenic variants in Stickler syndrome-associated genes and other genes. The onset age of the RRD in patients with stickler syndrome (35.17 ± 18.03 years old) was younger than others (49.35 ± 15.26 years old) (p = 4.77E-2). Moreover, the refractive error of the patients with Stickler syndrome is milder (−10.83 ± 2.62 VS −16.09 ± 4.87, p = 1.46E-2) and the axial length is shorter (27.63 ± 1.01 VS 29.54 ± 1.86, p = 1.92E-2) compared to other patients with high myopia and RRD (Figure 2).

## Discussion

Our study enrolled 40 patients with high myopia and concurrent RRD. Pathogenic variants were detected in 47.5% (19/40) of cases across 281 RetNet genes, 178 genes associated with syndromic high myopia, 23 genes linked to non-syndromic high myopia, and 29 genes responsible for RRD.

Variants in RetNet gene were the most common cause of RRD in patients with high myopia, accounting for 45.0% (18/40) of cases. Comparing with our previous study, pathogenic variants in RetNet genes were found in 23.4% of non-syndromic early-onset high myopia patients and 7.2% of non-syndromic late-onset high myopia patients (Zhou et al., 2018). The frequency of pathogenic variants in RetNet genes was significantly higher in patients with high myopia complicated by RRD compared to those with early-onset (18/40 VS 76/325, p = 3.18E-3) or non-syndromic late-onset high myopia (18/40 VS 14/195, p = 2.11E-10) without RRD. Although the RetNet database now includes 281 genes, an increase from the previous 234, the pathogenic variants identified in our study still correspond to the original 234 genes, rather than the newly added ones. Notably, the probands in the previous studies on early-onset and late-onset high myopia did not experience retinal detachment, which supports the validity of comparing these groups. Therefore, these results are directly comparable.

Genes associated with retinal detachment could account for 27.5% (11/40) of patients with high myopia complicated by RRD. This finding is consistent with the well-established association between myopia and an increased risk of RRD. Myopic individuals are more prone to posterior vitreous detachment, retinal tears, lattice degeneration, and retinal thinning (Mitry et al., 2011). These characters may reflect an inherent fragility of the retina, predisposing them to retinal detachment. Therefore, RRD is strongly correlated with high myopia.

Genes associated with syndromic high myopia accounted for 25.0% (10/40) of patients with high myopia complicated by RRD. No variants were detected in genes associated with non-syndromic high myopia. Syndromic high myopia includes high myopia accompanied by either ocular or systemic abnormalities, such as retinitis pigmentosa, congenital night blindness, Stickler syndrome, Marfan syndrome, Weill–Marchesani syndrome, Knobloch syndrome, Cohen syndrome, and Wagner syndrome. Notably, the most common pathogenic genes in high myopia cases with retinal detachment were those related to Stickler syndrome, particularly *COL2A1* (10.0%, 4/40) and *COL11A1* (5.0%, 2/40). *VCAN* related to Wagner syndrome accounted for 5.0% (2/40), while *RP1* associated with retinitis pigmentosa were found in 2.5% (1/40) of cases.

Genes associated with RRD accounted for 27.5% (11/40) of patients with high myopia complicated by RRD. To date, at least 29 genes have been implicated in retinal detachment (Govers et al., 2023). These include genes related to conditions such as Stickler syndrome, Kniest dysplasia, Marshall syndrome, Knobloch syndrome, Marfan syndrome, among others. The most extensively research has been conducted on genes associated with Stickler Syndrome (Moledina et al., 2022). Stickler Syndrome is a hereditary multisystem connective tissue disorder characterized by a spectrum of phenotypes, all associated with pathogenic variations in genes guiding the production of collagen proteins, specifically type II, XI, and IX. Pathogenic variations affecting these collagen proteins typically result in abnormalities in the eyes, facial features, hearing, and skeletal structure. Stickler Syndrome, primarily caused by variants in *COL2A1* and *COL11A1*, represents the most common form, accounting for approximately 80%–90% of cases (Hoornaert et al., 2010; Soh et al., 2022). Other pathogenic genes include *COL9A1*, *COL9A2*, *COL9A3*, and *LOXL3* (Nixon et al., 2022).

Approximately 60% of Stickler syndrome patients eventually develop retinal detachment, often resulting in severe visual impairment and long-term morbidity. Patients with retinal detachment typically require multiple surgical interventions, with a high recurrence rate, leading to an overall poor visual prognosis (Coussa et al., 2019; Ang et al., 2008). In our study, compared to patients with unknown or other pathogenic gene variants, those with Stickler-related gene variants had the youngest average onset age of retinal detachment (35.17 ± 18.03 years old) and shorter axial length (27.63 ± 1.01 mm). Given these severe outcomes, some experts advocate for prophylactic retinal laser treatment in Stickler syndrome patients to reduce or prevent future RRD, although conclusive evidence supporting the absolute benefits of such treatments remains limited (Naravane et al., 2022; Fincham et al., 2014).

In conclusion, our findings indicate that Stickler syndrome, accounting for 15.0% of cases, is a predominant cause of high myopia accompanied by RRD. RetNet genes were implicated in 45.0% of cases in this cohort. As ophthalmologists, we advocate for increased attention to comprehensive systemic and ophthalmic examinations in high myopia patients to facilitate early intervention and reduce the risk of retinal detachment.

## Data Availability

The raw sequence data reported in this study have been deposited in the Genome Sequence Archive (Genomics, Proteomics & Bioinformatics 2021) within the National Genomics Data Center (Nucleic Acids Res 2022), China National Center for Bioinformation / Beijing Institute of Genomics, Chinese Academy of Sciences (GSA-Human: HRA010850). These data are publicly accessible at the following link: https://ngdc.cncb.ac.cn/gsa-human.
